# Case report: ZEB1 expression in three cases of hepatic carcinosarcoma

**DOI:** 10.3389/fonc.2022.972650

**Published:** 2022-09-12

**Authors:** Mingming Zhang, Dongchang Yang, Lu Li, Lin Liu, Ting Wang, Tao Liu, Lei Li, Yanrong Liu

**Affiliations:** ^1^ Department of Pathology, Affiliated Hospital of Jining Medical University, Jining Medical University, Jining, China; ^2^ School of Clinical Medicine, Jining Medical University, Jining, China; ^3^ Department of Surgery, Affiliated Hospital of Jining Medical University, Jining Medical University, Jining, China; ^4^ Health Management Center, Affiliated Hospital of Jining Medical University, Jining Medical University, Jining, China

**Keywords:** hepatic carcinosarcoma, EMT, ZEB1, liver, carcinosarcoma

## Abstract

Hepatic carcinosarcoma (HCS) is defined as a tumor that contains cancer from the epithelium and sarcoma from mesenchymal tissue. HCS has a low incidence rate and is composed of osteosarcoma, chondrosarcoma, or angiosarcoma. Though surgery is the main treatment for HCS, it has proven unsatisfactory, resulting in a very poor prognosis of HCS. Currently, the reports on HCS are mainly about the description of clinical pathological phenomena, imaging features, and mutation sites of related genes, the underlying molecular mechanism of HCS remains undefined. Through the dynamic process of epithelial-mesenchymal transition (EMT), cancer cells acquire a mesenchymal phenotype, simultaneously losing epithelial properties. Zinc finger E-box binding homeobox 1 (ZEB1) is an EMT-inducing transcription factor; its main regulatory target is E-cadherin in EMT process. Esophageal carcinosarcoma (ECS) is associated with EMT. The current study showed that EMT might promote the development of ECS and uterine carcinosarcoma (UCS), and ZEB1 was highly expressed in the sarcomatous components. In the current study, three cases were collected, and the clinicopathological features were compared with those of corresponding cases. The expression level, and subcellular localization of ZEB1 were detected using immunohistochemistry. The expression of the ZEB1 in the nucleus was found to be significantly higher in sarcomatous components than that in cancer components in all three cases, suggesting an association of HCS with EMT.

## Introduction

Hepatic carcinosarcoma (HCS), a rare malignant tumor, combines carcinomatous and sarcomatous elements (1). The tumor comprise spindles or polymorphous tumor cells with a mesenchymal character and polygonal cancer cells with epithelioid morphology (2). Usually manifesting in advanced stages, HCS demonstrates aggressive behavior and has a poor prognosis (3). Other than curative primary resection, no effective treatment options exist for HCS. Several studies have shown concordant genomic alterations in microdissected carcinomatous and sarcomatous components of HCS, strengthening the notion of a monoclonal origin of HCS (4). Current research on HCS remains predominantly limited to the description of clinicopathological phenomena, the molecular mechanism underlying HCS remains unclear.

Several studies have proved that in epithelial-mesenchymal transition (EMT), the histological change from epithelial elements to mesenchymal elements plays a vital role in the progression and metastasis of various cancers (5, 6). The hallmark of the EMT is the loss of epithelial surface markers, most remarkably E-cadherin (E-cad), and the acquisition of mesenchymal markers, including vimentin (Vim) and N-cadherin(N-cad) (7, 8). Carcinosarcoma (CS) possesses both epithelial and mesenchymal components and expresses several markers of EMT, including E-cad and Vim, suggesting HCS to be a prototype of EMT-related neoplasia. EMT is associated with the tumorigenesis of uterine carcinosarcoma (UCS) and esophageal carcinosarcoma (ECS) (9, 10). The association of HCS with EMT remains unclear.

In this study, three cases were collected, and the expression level of ZEB1 and its subcellular localization was detected by immunohistochemistry (IHC). The expression level of ZEB1 was found remarkably higher in the nucleus of the sarcomatous components than that in cancer components, suggesting the role of ZEB1 in regulating HCS *via* EMT.

## Methods

### Patients and data collection

Three cases were collected from the Affiliated Hospital of Jining Medical University from January 2013 through September 2021. The parameters of blood samples of each case were summarized, including serum levels of hepatitis B surface antigen (HBsAg), carcinoembryonic antigen (CEA), carbohydrate cancer antigen 19-9 (CA19-9), and alpha-fetoprotein (AFP). The HCS samples were biopsied, formalin-fixed, and paraffin-embedded. All the patients signed the prior informed consent. The Ethics Committee of the Affiliated Hospital of Jining Medical University granted the necessary ethical approval for this study.

### Immunohistochemistry analysis

The tissues of the sample cases were deparaffinized with xylene and dehydrated with ethanol at decreasing concentrations. Endogenous peroxidase was blocked by incubating with 3% hydrogen peroxide for 15 min. After incubation with normal goat serum for 20 min at room temperature to block unspecific labeling, the tissues were incubated with primary antibodies (**Table S1**) in a humidified chamber overnight at 4°C. Diaminobenzidine was used for color development, and hematoxylin was used as a counterstain. The staining index of ZEB1 was evaluated. A staining index was used to interpret the results by analyzing both the staining intensity and the proportion of positive cells (11). The staining index (value, 0-12) was determined by multiplying the score for staining intensity with the score for the positive area. The staining index was evaluated by two pathologists. The ZEB1 staining index of the nucleus and cytoplasm was counted in 200× separately, and the average of five fields was determined. The ZEB1 staining index of the nucleus and cytoplasm in cancer cells and sarcomatous cells were respectively analyzed.

### Statistical analysis

Statistical analyses were performed using GraphPad Prism (GraphPad Software, Inc., USA) and SPSS version 17.0 (SPSS Software, USA). Data from biological experiments were presented as mean ± standard deviation. Two-tailed unpaired Student’s t-test was used to compare the data of the two groups. Differences were considered statistically significant when *P* < 0.05.

## Results

### Patient characteristics

**Table 1** shows the characteristics of the patients with HCS. Case 1 was male, and cases 2 and 3 were female. The mean age was 64 years. The main symptoms reported were epigastric discomfort in all cases. All patients underwent computed tomography (CT) and radical surgery with complete tumor resection. During the operation, the tumor was found invading the right posterior diaphragm and perirenal fat sac in case 2. In case 3, the tumor had severe adhesion with the omentum and right colon, and the tumor ruptured during surgical resection.

**Table 1:** Clinical characteristics of the three patients with HCS.

	Case 1	Case 2	Case 3
Gender	Male	Female	Female
Age	64	66	64
Early symptoms	Epigastric discomfort	Epigastric discomfort	Epigastric discomfort
Tumor examination	15x11cm	7x6.5cm	13x6.7cm
Location in the liver	Right	Right	Right
Serum AFP (normal < 20μg/l)	3.47	24.87	340.28
Serum CEA (normal < 10μg/l)	1.68	1.51	2.15
Serum CA19-9 (normal < 39μg/l)	6.00	119.86	6.35
Serum albumin (normal 34-48 g/l)	49.10	32.90	31.6
Serum A/G (normal 1.5-2.5)	1.00	1.40	1.5
Serum ALT (normal 0-41 U/I)	32.30	26	28
Serum AST (normal 0-37 U/l)	25	24	43
Bold HBsAg (normal; negative)	Positive	Negative	Positive
Cirrhosis	No	No	No
Surgical method	Right hepatic tumor resection	Right hepatic tumor resection	Right hepatic tumor resection
Clinical diagnosis	HCC	HCC	HCC

AFP, alpha-fetoprotein; A/G, specific value of albumin and globulin; ALT, alanine aminotransferase; AST, serum aspartate aminotransferase; CEA, carcinoembryonic antigen; CA19-9, cancer antigen 19-9; HBsAg, hepatitis B surface antigen; HCC, hepatocellular carcinoma.

**Figure 1:**
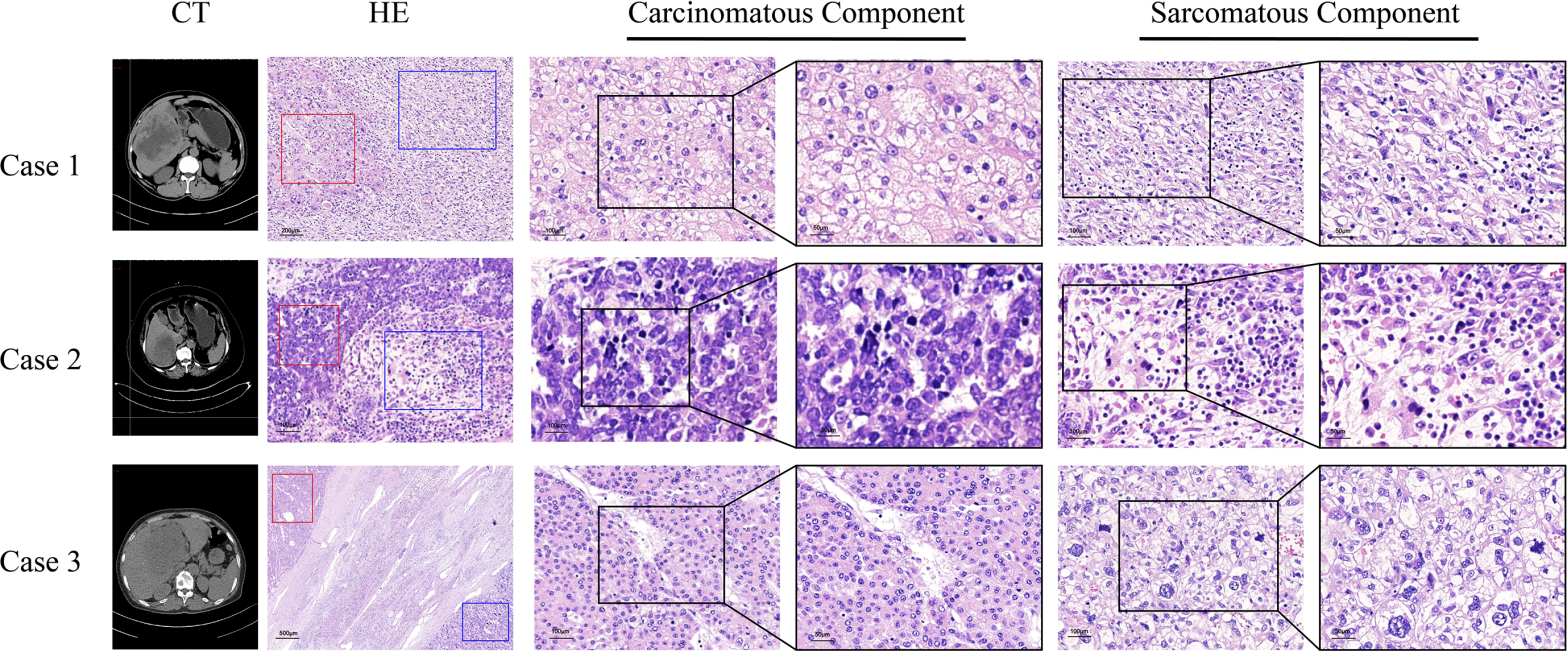
Computed tomography scans, the morphology of sarcomatous components and carcinomatous components in the tissues of three HCS cases. Red frames represent carcinomatous components and blue frames represent sarcomatous components.

CT images of the three patients are shown in **Figure 1** and **Supplementary Figure 1**. HCS usually showed low density in non-enhanced CT, heterogeneous enhancement in the arterial phase, and gradually decreased enhancement in the portal vein phase and in the delayed phase. Tumor boundaries are usually clear after enhancement. Because of the lack of specific radiological features, sometimes distinguishing HCS from hepatocellular carcinoma or hepatic abscess poses a challenge. In case 1, HCS showed a huge mixed-density space-occupying lesion in the right lobe of the liver. The tumor was unevenly enhanced in the arterial phase, and the enhancement degree in the portal vein phase and the delayed phase gradually decreased. In case 2, HCS showed an irregular mass with mixed cystic and solid components. Post enhancement, the solid components were enhanced unevenly and slightly in the arterial phase, though no obvious enhancement was found in the cystic area. The enhancement degree in the portal vein phase and delayed phase showed a gradual decrease. In case 3, the tumor showed a mixed density lesion in the right lobe, enhanced unevenly in the arterial phase and portal phase, and the degree of enhancement got decreased in the delayed phase. No enlarged lymph nodes or distant metastases were observed in all cases.

Cases 1 and 3 were found positive for HBsAg. The serum tumor biomarkers CEA, the serum CA19–9, and the serum AFP were negative in case 1. However, the serum AFP was found elevated in cases 2 (24.87 µg/L) and 3 (340.28 µg/L); the serum CA19–9 was observed to be elevated in case 2 (119.86 µg/L). All three cases underwent partial excision of the right hepatic lobes (**Table 1**).

### Pathological features

The tumor sizes of the three cases were 15.0 cm × 11.0 cm, 7.0 cm × 6.5 cm, and 13.0 cm × 6.4 cm, respectively. Histopathological findings indicated the existence of necrosis in the carcinomatous areas and sarcomatous areas in the three cases (**Table 1**). The sarcomatous cells exhibited spindle-shaped heterotypic cells with varying sizes of enlarged nuclei. The cancer cells showed polygonal epithelial phenotype. Both components were separate in certain areas and intermingled in other areas. In case 1, well-differentiated cancer cells were arranged in a trabecular pattern, the cytoplasm of cancer cells was eosinophilic, and some cells showed hyaline degeneration. In case 2, medium-differentiated cancer cells were arranged in the gland; a pseudo glandular structure was formed. In case 3, poorly differentiated cancer cells exhibited a nodular growth pattern. Numerous tumor giant cells and thick, irregular capillaries could be found in the sarcomatous areas. A clear boundary between the two components was observed. Besides, intravascular cancer emboli was noticed in case 3 (**Figure 1**).

### Molecular markers of HCS

**Figure 2:**
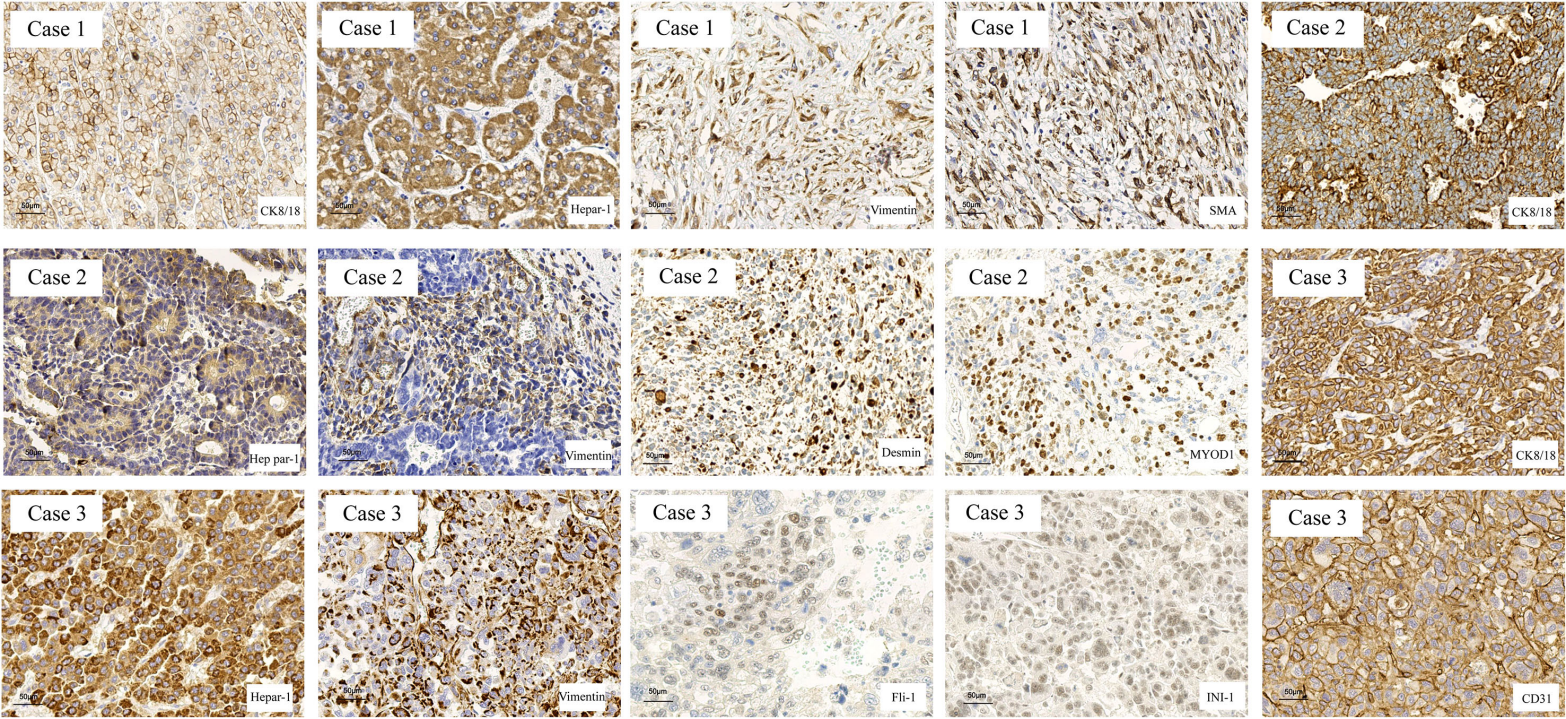
The markers of hepatocarcinoma and sarcoma were determined in HCS tissues by IHC. The hepatocarcinoma markers, including CK8/18 and Hep Par-1, were positive in the carcinomatous components, and the mesenchymal markers, including Vimentin, SMA, MYOD1, FLI-1, INI-1, and CD31, were used to determine the tissue source of the mesenchymal components.

When the three cases of HCS were examined immunohistochemically (**Figure 2**), all sarcomatous components were found positive for Vim staining but negative for CK8/18 and Hep Par-1. In contrast, all cancer components were found positive for CK8/18 and Hep Par-1 but negative for Vim. In case 1, the sarcomatous component was positive for smooth muscle actin (SMA), indicating its origin from smooth muscle differentiation. In case 2, the sarcomatous component was found positive for myogenic differentiation 1 (MyoD1), indicating its origin from striated muscle differentiation. In case 3, the sarcomatous component was positive for FLI1, INI-1, and CD31, indicating that it was derived from angiogenic differentiation.

### ZEB1 expression in HCS

**Figure 3:**
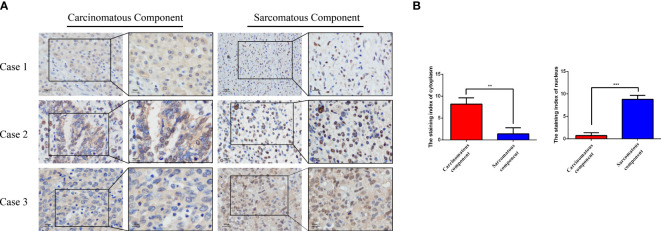
The expression of ZEB1 was examined in HCS tissues using IHC analysis. **(A)** Representative images of ZEB1 expression in the sarcomatous components and the carcinomatous components as detected by IHC analysis. **(B)** Statistical analysis of ZEB1 staining index in the sarcomatous components and the carcinomatous components of HCS tissues.**P < 0.01, ***P < 0.001.

Because the sarcomatous components were found positive for Vim, an EMT marker, we speculate the association of EMT with the tumorigenesis of HCS. Therefore, we evaluated the expression of ZEB1 by IHC, in the three cases of HCS (**Figure 3A**). In the carcinomatous components, the cytoplasmic staining index of ZEB1 was 8.200 ± 1.400 (mean ± SD; N=3); the nucleus staining index of ZEB1 was 0.7333 ± 0.6429 (mean ± SD; N=3). In the sarcomatous components, the cytoplasmic staining index of ZEB1 was 1.400 ± 1.400 (mean ± SD; N=3), and the nucleus staining index of ZEB1 was 8.800 ± 0.8718 (mean ± SD; N=3). The expression of ZEB1 is higher in the nucleus of the sarcomatous cells than that in cancer cells (*P* < 0.001), In contrast, a decrease in ZEB1 expression could be observed in the cytoplasm of the sarcomatous cells (*P* < 0.01) (**Figure 3B**). ZEB1 was found mainly expressed in the cytoplasm of the cancer cells, though primarily located in the nucleus of the sarcomatous cells. An increasing expression of ZEB1 was noted in the nucleus of the sarcomatous cells, suggesting the role of ZEB1 as a transcription factor in the nucleus; it also suggests an association of tumorigenesis of HCS with EMT.

## Discussion

CS is defined as having both definite cancer components and sarcoma components in the same tumor. The common incidence sites are lung, esophagus, breast, and so on. Primary carcinosarcoma of the liver is a rare occurrence. In 2010, World Health Organization (WHO) classified HCS as a type of mesenchymal tumor of the liver and defined it as a malignant tumor mixed with cancer-like components both hepatocyte-derived and cholangiocyte-derived and sarcoma-like components. In 2019, the WHO divided liver tumors into benign hepatocellular tumors, malignant hepatocellular tumors, and precancerous lesions. However, it does not include the classification of HCS. Currently, the main treatment interventions include surgical resection and postoperative chemotherapy. By using targeted next-generation sequencing, studies have revealed frequent oncogenic aberrations involving tumor protein p53 (TP53), NF1/2, and vascular endothelial growth factor A (VEGFA) in both tumor components, suggesting their correlation with HCS (4). The tumorigenesis of HCS remained poorly understood.

Most of the studies on CS focus on the description of clinical pathological phenomena though a few studies focus on the mechanism of tumorigenesis of CS. Sarcoma components may derive from EMT in CS (12). ALK-related signal cascades may participate in initial signaling for sarcomatous differentiation driven from carcinomatous components through the induction of the EMT process of UCS (13).

ZEB1, a crucial EMT transcription factor, is a member of the E-box binding zinc finger protein family of zinc finger structure transcription factors and is necessary for embryonic development. The zinc finger structure is a common DNA binding unit in eukaryotic cells. The ZEB family contains ZEB1 and ZEB2 protein transcription factors. ZEB1 gene expression is highest in the bladder and uterus in normal tissues and highest in the heart, lung, and thymus during embryonic development (14). A few studies have indicated that ZEB1 is closely related to tumorigenesis and tumor invasion (15, 16). A high level of ZEB1 expression is correlated with poor outcomes, including chemotherapy resistance (17). In ECS, ZEB1 has been suggested to play a critical role in the EMT process (10). A significantly higher expression of ZEB1 was observed in the sarcomatous components in UCS (18). However, the role of ZEB1 as a key transcription factor in HCS remains unclear.

The current study presents the clinicopathological and radiological features of HCS. Further, the ZEB1 staining index in the nucleus was found to increase in the sarcomatous components of HCS than that in the carcinomatous component. The study results revealed that ZEB1 is highly characteristic of sarcoma components of EMT, suggesting an association of tumorigenesis of HCS with EMT. Overall, more cases are still needed to verify the correlation between HCS and EMT.

## Data availability statement

The original contributions presented in the study are included in the article/**Supplementary Material**. Further inquiries can be directed to the corresponding authors.

## Ethics statement

Written informed consent was obtained from the individual(s) for the publication of any potentially identifiable images or data included in this article.

## Author contributions

MZ and DY performed the experiments, DY performed data collection, and MZ performed data analysis. TL and LuL contributed to the collection of clinical data. YL and LeL reviewed the pathological sections. MZ wrote the manuscript; TW and YL designed the study. YL, TW and LiL revised the manuscript. All authors contributed to the article and approved the submitted version.

## Funding

This work was supported by the National Natural Science Foundation of China (81972629), the Taishan Scholars Program of Shandong Province (tsqn201909193), Shandong Youth Innovation and Technology program (2020KJL003).

## Conflict of interest

The authors declare that the research was conducted in the absence of any commercial or financial relationships that could be construed as a potential conflict of interest.

## Publisher’s note

All claims expressed in this article are solely those of the authors and do not necessarily represent those of their affiliated organizations, or those of the publisher, the editors and the reviewers. Any product that may be evaluated in this article, or claim that may be made by its manufacturer, is not guaranteed or endorsed by the publisher.
