# Supplementary figures and images for: Case report: ZEB1 expression in three cases of hepatic carcinosarcoma
Source: Front Oncol. 2022 Sep 12;12:972650. doi: 10.3389/fonc.2022.972650 (PMC9511137; doi:10.3389/fonc.2022.972650)

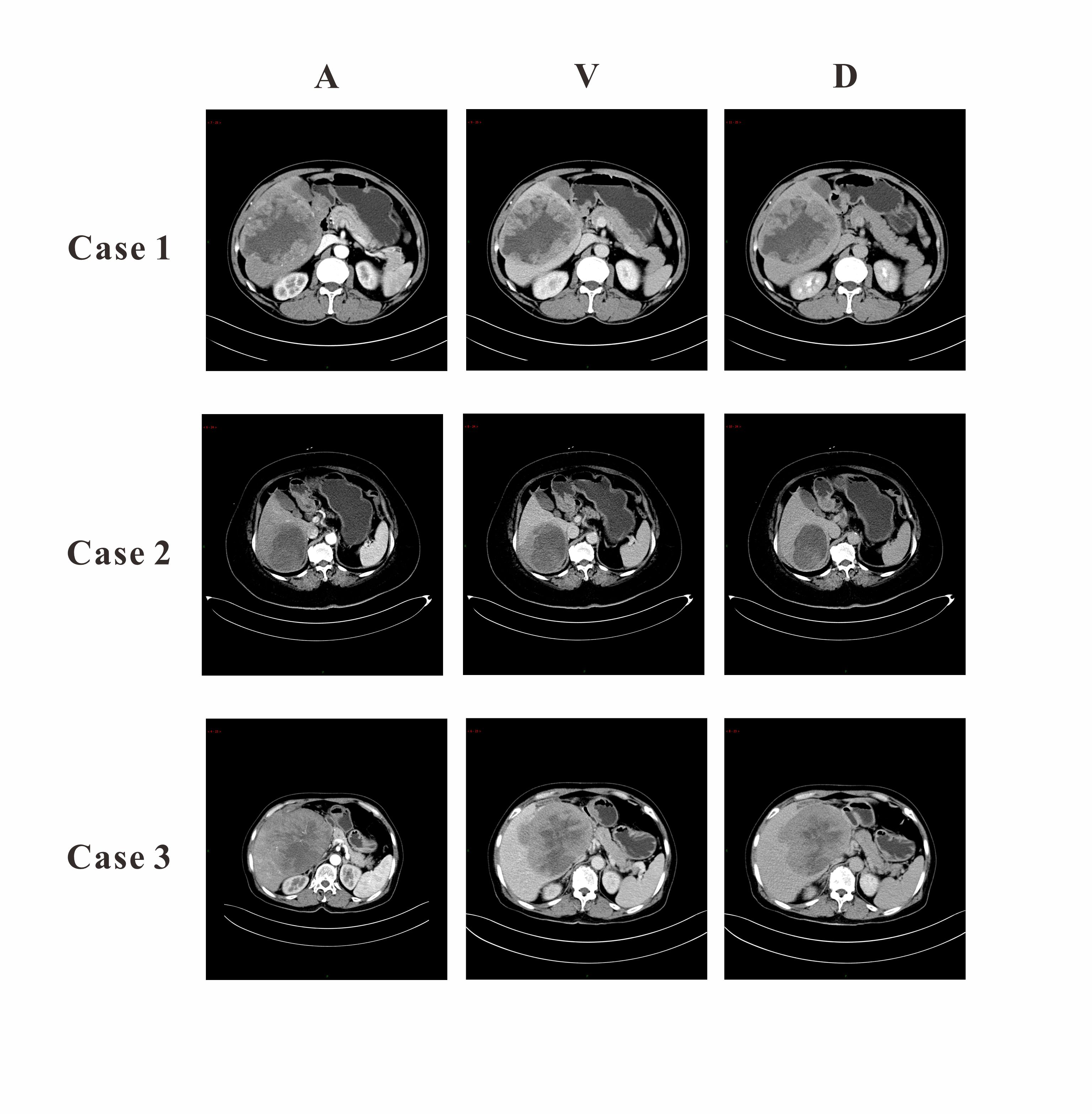

Supplement: Supplementary file 1 [file Image_1.jpeg]
