# Supplementary material for: Case report: ZEB1 expression in three cases of hepatic carcinosarcoma
Source: Front Oncol. 2022 Sep 12;12:972650. doi: 10.3389/fonc.2022.972650 (PMC9511137; doi:10.3389/fonc.2022.972650)
Supplement: Supplementary file 2 [file Table_1.doc]

Table S1 Primary Antibodies used for IHC

| Antibody | Company | Catalogue Number | Type | Clone | Dilution |
| --- | --- | --- | --- | --- | --- |
| CK8/18 | ZSGB-BIO | ZM-0315 | Mouse monoclonal | B22.1&B23.1 | Working Fluid |
| Hep Par1 | ZSGB-BIO | ZM-0131 | Mouse monoclonal | Hep Par1 | Working Fluid |
| Vimentin | ZSGB-BIO | ZM-0260 | Mouse monoclonal | UMAB159 | Working Fluid |
| SMA | ZSGB-BIO | ZM-0003 | Mouse monoclonal | UMAB237 | Working Fluid |
| Desmin | ZSGB-BIO | ZA-0610 | Mouse monoclonal | EP15 | Working Fluid |
| Myo D1 | ZSGB-BIO | ZA-0585 | Mouse monoclonal | ZA-0585 | Working Fluid |
| Fli-1 | ZSGB-BIO | ZM-0108 | Mouse monoclonal | G146-22 | Working Fluid |
| INI-1 | ZSGB-BIO | ZM-0173 | Mouse monoclonal | ZM-0173 | Working Fluid |
| CD31 | ZSGB-BIO | ZM-0044 | Mouse monoclonal | UMAB30 | Working Fluid |
| ZEB1 | Abcam | ab181451 | Mouse monoclonal | 2A8A6 | 1:200 |
